# The Timing of the Shrew: Continuous Melatonin Treatment Maintains Youthful Rhythmic Activity in Aging *Crocidura russula*

**DOI:** 10.1371/journal.pone.0005904

**Published:** 2009-06-15

**Authors:** Elodie Magnanou, Joël Attia, Roger Fons, Gilles Boeuf, Jack Falcon

**Affiliations:** 1 UPMC University of Paris 06, UMR 7628, Banyuls/Mer, France; 2 CNRS, GDR 2821 and UMR 7628, Banyuls/Mer, France; 3 University Jean Monnet, Écologie et Neuro-Ethologie Sensorielle, Saint-Étienne, France; 4 Museum National d'Histoire Naturelle, Paris, France; Pennsylvania State University, United States of America

## Abstract

**Background:**

Laboratory conditions nullify the extrinsic factors that determine the wild expected lifespan and release the intrinsic or potential lifespan. Thus, wild animals reared in a laboratory often show an increased lifespan, and consequently an increased senescence phase. Senescence is associated with a broad suite of physiological changes, including a decreased responsiveness of the circadian system. The time-keeping hormone melatonin, an important chemical player in this system, is suspected to have an anti-aging role. The Greater White-toothed shrew *Crocidura russula* is an ideal study model to address questions related to aging and associated changes in biological functions: its lifespan is short and is substantially increased in captivity; daily and seasonal rhythms, while very marked the first year of life, are dramatically altered during the senescence process which starts during the second year. Here we report on an investigation of the effects of melatonin administration on locomotor activity of aging shrews.

**Methodology/Principal Findings:**

1) The diel fluctuations of melatonin levels in young, adult and aging shrews were quantified in the pineal gland and plasma. In both, a marked diel rhythm (low diurnal concentration; high nocturnal concentration) was present in young animals but then decreased in adults, and, as a result of a loss in the nocturnal production, was absent in old animals. 2) Daily locomotor activity rhythm was monitored in pre-senescent animals that had received either a subcutaneous melatonin implant, an empty implant or no implant at all. In non-implanted and sham-implanted shrews, the rhythm was well marked in adults. A marked degradation in both period and amplitude, however, started after the age of 14–16 months. This pattern was considerably delayed in melatonin-implanted shrews who maintained the daily rhythm for significantly longer.

**Conclusions:**

This is the first long term study (>500 days observation of the same individuals) that investigates the effects of continuous melatonin delivery. As such, it sheds new light on the putative anti-aging role of melatonin by demonstrating that continuous melatonin administration delays the onset of senescence. In addition, the shrew appears to be a promising mammalian model for elucidating the precise relationships between melatonin and aging.

## Introduction

Senescence is defined as the increase in the rate of mortality and the decrease of reproductive success as a function of age [1], [2]. This phenomenon is the result of a complex combination of physiological changes that occur at the level of the whole organism as well as individual organs and tissues [3]. In mammals, these changes lead to a decreased responsiveness to the phase-shifting effects of light stimuli which is characterized by an alteration of the sleep/wake pattern and a decrease in daytime alertness and cognitive performance. Although little is known about the precise physiological mechanisms underlying normal age-related changes in biological timing, it has been suggested that they reflect changes in the suprachiasmatic nucleus (SCN), the neural structure thought to be primarily responsible for generation of the circadian oscillation [4]. The SCN of the hypothalamus constitutes the master circadian pacemaker whose activity is synchronized by the photoperiodic information perceived through the eyes. One SCN output pathway ends at the pineal gland and induces the rhythmic (nocturnal) production of melatonin [5]. The time-keeping properties of melatonin result in part from feed-back action on the SCN. Melatonin production decays during aging (reviewed in [6], [7]). Pinealectomy leads to an acceleration of many aspects of the aging process which can be partially reversed or reduced by melatonin treatment [8]–[10]. This set of clues indicates that the hormone might play an anti-aging role [11]. Until now, studies aimed at understanding the specific effects of melatonin on age-related deterioration of the circadian rhythm were based on short-term acute melatonin administration [12]–[14]. As a result, it remains unclear if the anti-aging effect of melatonin on the time-keeping system is due to its rhythmic production or to its production *per se*.

As in all shrews, Greater White-toothed shrew *Crocidura russula* (Hermann, 1780) physiology is notable for extremely rapid gas exchange. Its mass-specific metabolic rate is among the highest ever measured in terrestrial mammals [15]–[19]. As predicted by the rate-of-living theory [20] (the higher the energy expenditure, the shorter the lifespan), *C. russula*'s lifespan is only 18 months in the wild [21] but it can be increased up to 30 months under laboratory conditions [22]. *C. russula* exhibits seasonal and daily rhythmic components in most of its biological functions including reproduction, thermoregulation, coat replacement and locomotor activity [19]. Shrews maintain their daily and seasonal rhythms up to one year after weaning. At that point, senesence related processes such as arrhythmic daily locomotor activity, absence of reproduction and erratic shedding begin to appear [23]. Despite the increased lifespan under laboratory conditions, the aging process in captive animals starts at the same point as in wild animals. Because of its extreme metabolic rate, its pronounced biorhythmicity and its short lifespan, *C. russula* is an ideal study model to investigate the possible effects of melatonin on the deterioration of biorhythms due to senescence.

To determine whether it is the rhythmic administration of melatonin or the administration itself that impacts traits related to aging, we investigated the effect of a long-term continuous melatonin delivery on adults *C. russula*. We monitored the daily activity rhythms of animals starting just before the first clues of senescence appeared until death, *i.e*., over a period of more than 500 days. To the best of our knowledge, this is the first time that such a long-term survey has been performed. We show that a continuous delivery of melatonin through subcutaneous implants, maintains the rhythmic activity pattern in aging animals for an additional year when compared to controls.

## Results

### Melatonin measurements

#### Pineal gland and plasma melatonin concentrations

**Figure 1:**
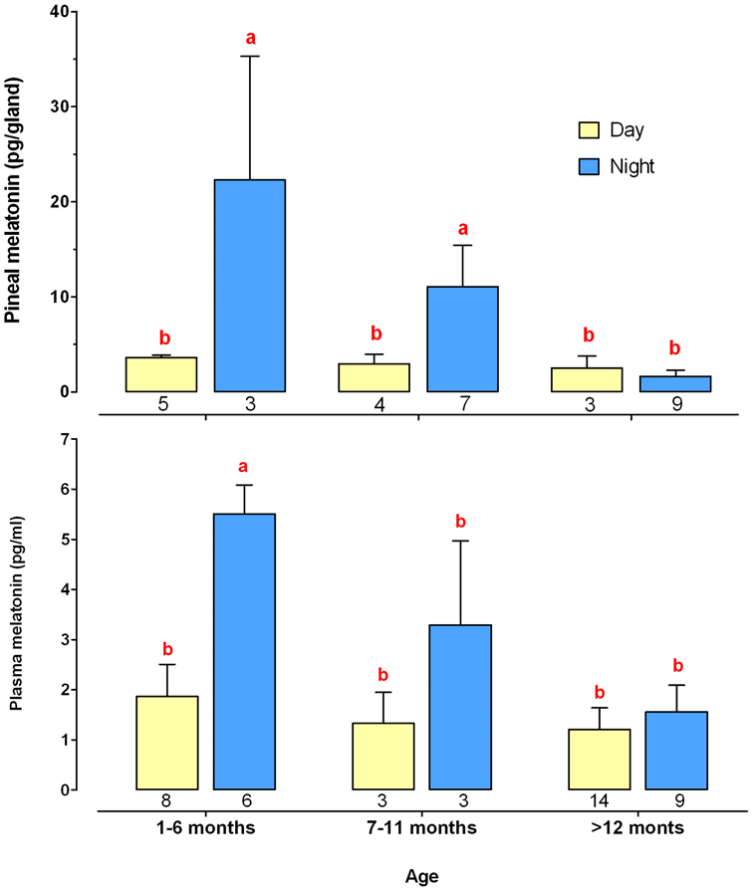
Day/night variations in pineal and plasma melatonin in shrews from different ages. Shrews were grouped as young (<7 mo), adult (7–12 mo) and aged (>12 mo) as indicated on the abscissa. Values are expressed as the mean±Standard Error of the Mean (SEM). The number of individuals is indicated below the corresponding column. There was a significant effect of age on both pineal gland and plasma melatonin content (SRH test: *P* = 0.001 and *P* = 0.003 respectively). Pineal gland and plasma concentrations of young and adult animals were significantly different between night and day, but this marked diel rhythm disappeared in old animals as a result of a loss in the nocturnal production (different letters indicate significant differences, Mann-Whitney U test, *P*<0.05).

A similar pattern of melatonin production was observed in the pineal gland and plasma (sample size of respectively 31 and 44) (Fig. 1). Night-time melatonin concentrations decreased significantly with age, whereas daytime levels were not affected and remained low lifelong. A daily pattern was evident for only up to 12 month old (mo) shrews (Fig. 1), although nocturnal levels were lower in adult (6–12 mo) *vs*. young (<6 mo) shrews. In aged (>12 mo) shrews, nocturnal melatonin levels were similar to diurnal levels both in the pineal gland and plasma.

#### Melatonin release by the implant

**Figure 2:**
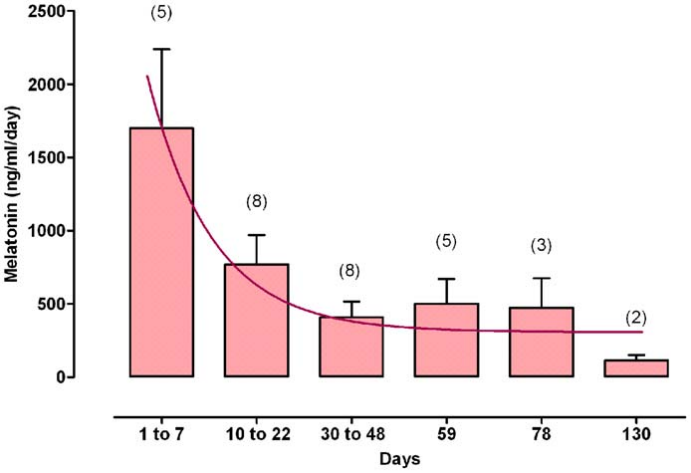
24 h Melatonin release by the implant. Melatonin implants were individually immersed in 1 mL physiological buffer renewed every 24 hours for up to 130 days. Melatonin concentration measured in the physiological serum are the Mean±SEM (n is indicated under brackets). The data fitted an exponential decay.

Melatonin measurements were made in four implanted shrews in order to estimate the levels induced by the implant. Plasma levels averaged 50 ng/ml one week after implantation (2 animals), and decreased to 120 pg/ml 3 months later (2 animals). In the *in vitro* system, melatonin release diminished with time, fitting an exponential decay (Fig. 2). High at the beginning of the experiment, it decreased rapidly during the first ten days and then remained within the same order of magnitude until the end of the survey.

### Diel locomotor activity rhythm

**Figure 3:**
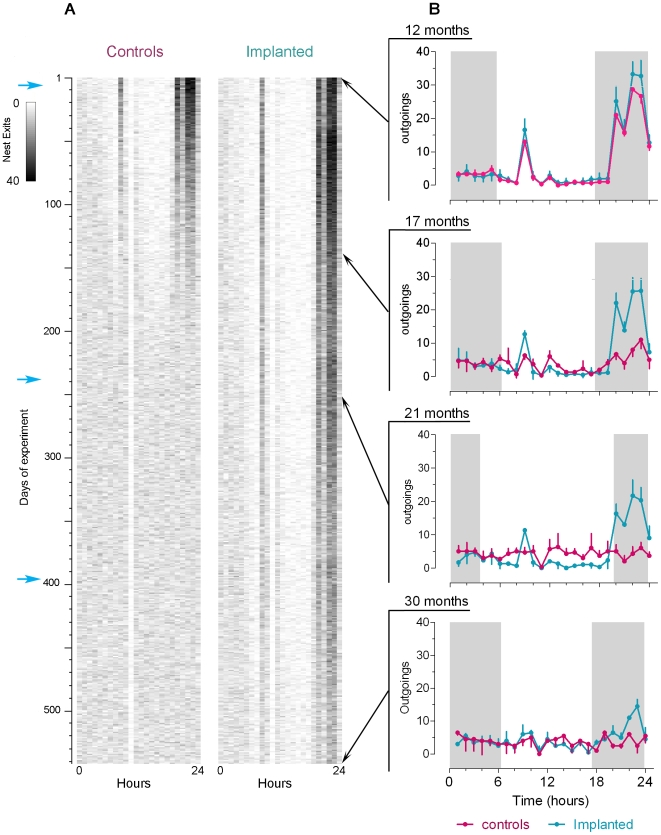
Shrews' locomotor activity over the duration of the experiment. A: Actograms of a control and a melatonin-implanted shrew from the age of 12 to 30 months. Each line corresponds to one experimental day and there are 24 boxes per line (one box per hour). Nest exits are expressed as a grey scale: the darker the area, the higher the activity for a particular period. Blue arrows indicate each time a new implant was supplied. B: Daily activity profiles (mean±SEM) of controls (red) and melatonin-implanted (blue) shrews at, respectively, 12, 17, 21 and 30 months after the beginning of the experiment. Shrews were submitted to a natural light–dark regime. Grey boxes correspond to the night. Due to mortality, the number of individuals decreased during the experiment so that n was 11, 9, 6 and 4 at respectively month 12, 17, 21, and 30.

**Figure 4:**
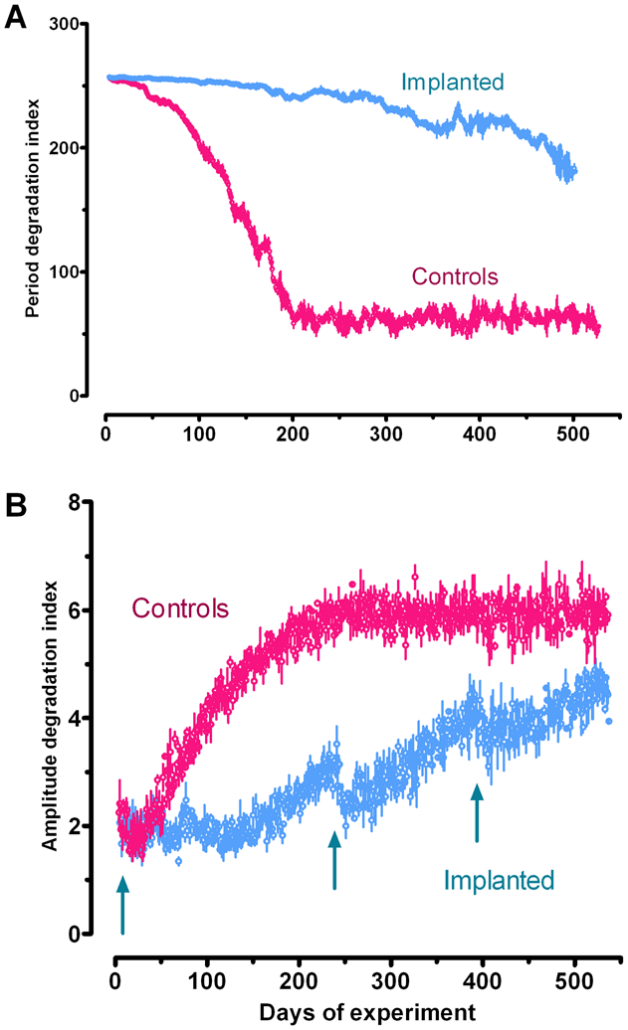
Period and amplitude degradation indexes over the duration of the experiment. For each individual, the 24 h period (A) and amplitude (B) degradation indexes were calculated as explained in the text, and are reported as a function of the time course of the experiment. The blue arrows indicate each time a new implant was supplied. Values were calculated from the data presented in Figure 3.

All individuals, aged 11–15 mo, displayed a marked diel activity rhythm, which was the highest during the first half of the night (expressed as the number of nest exits per hour) (Fig. 3). A secondary smaller peak was observed early during the light phase (Fig. 3). This bimodal pattern had a marked 24 h periodicity of high amplitude. After month 15, the amplitude of the rhythm decreased dramatically in both controls and sham-implanted shrews, and was no longer observed after month 17 (Figs 3, 4). In parallel, the significance of the 24 h period also decreased to the point where it was no longer discernable (Figs 3, 4). In contrast, in the implanted animals (i) the first noticeable decrease in amplitude of the rhythm was delayed by 3 months and (ii) the 24 h period remained highly significant until death (Figs 3, 4). In addition, the second and, to a lesser extent, the third implants induced a temporary gain in amplitude (Fig. 4B).

## Discussion

This study provides new and important data on the role of melatonin in both the regulation of clock-controlled processes and aging. The continuous monitoring of spontaneous locomotor activity has emerged as one of the most widely used behavioral outputs in circadian research. More specifically, it is considered as extremely useful for the description of the effects of aging on circadian rhythms [24]. Here we report on the first long term (>2 years) investigation of the effects of a continuous melatonin treatment on the behavior of a vertebrate.

### Age-related disruption of the melatonin rhythm

This is the first set of experiments aimed at characterizing the diel pattern of melatonin secretion in the shrew. Two main characteristics were found. First, in untreated animals, pineal gland and plasma variations in melatonin content were strongly correlated. Second, in agreement with studies of other vertebrates [25], [26], both profiles changed dramatically during the course of the shrews' life. The diel rhythm was robust in young animals (<7 mo), diminished in adults (7–12 mo), and absent in aged shrews >12 mo). This resulted from a progressive decrease in the nocturnal production. These data fit with the known biology of the *C. russula* and its lifespan which is only 18 months in the wild [21]. It is a well known phenomenon that aging results in decreased nocturnal melatonin production and deterioration of the daily rhythmic pattern [7], [27]–[31]. The reasons are yet unknown, and may involve a disruption at some step of the circadian pathway that controls the nocturnal surge in melatonin secretion [32]. Degenerative changes are observed in the optic nerve and retina of aging humans [33]. Aging may impair the sensitivity of the SCN to retinal input [9], [30]. A compelling set of studies also reports a deterioration within the clock mechanism of the SCN itself [7], [9], [30], [34]. Finally, it is possible that the decline in melatonin production with age is a consequence of a deficit at the level of the pineal gland. As suggested elsewhere [6], reduced activity of the arylalkylamine *N*-acetyltransferase, linked to an impaired pineal catecholaminergic neurotransmission, could result in diminished or suppressed nocturnal production. Moreover, the aging process leads to pineal gland calcification [35], [36]. Our investigations (unpublished) have shown modifications at the level of the pinealocytes and an accumulation of calcium deposits in both the pineal organ and retina of aged shrews compared to young individuals. Altogether, these data indicate that shrews older than 12 months have entered the aging process. This conclusion is supported by our studies on the locomotor activity, as discussed below.

### Age-related disruption of locomotor activity rhythm

Adult shrews (12 mo) exhibited a significant bimodal diel pattern of activity of marked amplitude. We found that the amplitude of the rhythm decreased dramatically in controls and sham-implanted shrews, starting the second year of life, such that no rhythm was apparent after month 17. This is consistent with observations made on another Crocidurinae [37]. This pattern was parallel to the decrease of pineal gland and plasma melatonin levels described above. Whether there is a causal relationship between the age-related loss in melatonin production and the response to photoperiod [7] remains an open issue. It is interesting to note that the melatonin rhythm does disappear in healthy old men, except centenarians [31], [38]. Understanding what factors underlie the disruption of the circadian system, including melatonin production, remains an issue of considerable interest [24], [34]. In this regard, the continuous monitoring, over more than 500 days, of the spontaneous locomotor activity of *C. russula*, turns out to be extremely useful for the characterization of the effects of aging on daily rhythms. This unique system provides original and interesting data on the effects of melatonin on the age-related loss of locomotor activity rhythm.

### Melatonin treatment and locomotor activity rhythm

Insertion of a subcutaneous melatonin implant induced a dramatic increase in plasma melatonin level in shrews where it was measured. At one week and three months after implantation, plasma melatonin levels were ≈5000-fold and 20-fold, respectively the nocturnal levels of control shrews. Assuming a half life of ≈20 min for plasma melatonin [39], it seems that the values found in the *in vitro* system (as expressed per day in Fig. 2) are within the range of those measured in the plasma of the implanted animals. This would indicate that there was an extremely high release over the first 10 days, followed by a stabilization to lower values which nevertheless remained high compared to melatonin concentrations quantified in the plasma of non-implanted animals. It is therefore clear that we are dealing here with pharmacological levels delivered on a continuous basis.

Previous studies had tested the effects of melatonin on age-related decreases in daily activity rhythms. However, these were short-term studies that delivered melatonin on an acute or chronic basis [12]–[14]. The present study emphasizes that a treatment delivering high doses of melatonin continuously does not suppress the diel activity pattern in adult shrews. On the contrary, the rhythm of 12 mo individuals was reinforced. Furthermore, the long-term treatment slowed the age-induced loss of shrew rhythmic behavior. It is notable that melatonin does not need to be delivered on a rhythmic basis in order to allow maintaining the synchronization of the daily activity pattern. This suggests that constant delivery of the hormone, when provided before the onset of senesence, compensates for the lack of production by aged animals. As discussed above for melatonin, the degradation of the daily rhythm in the aging shrew probably reflects changes in the circadian system that controls it. Alterations are likely to affect the pacemaker mechanism itself [40]. It is possible that the effects of melatonin observed here are due to a protective action on the SCN, perhaps by taking over the natural production that normally decreases with age. It has been suggested though that not all clock stimuli lose their effectiveness with age and that it may be possible to compensate for deficits in clock performance by enhancing the strength of those stimulus pathways which are still intact [40]. A recent model of the circadian clock suggests that outputs of the clock which also feed back into it, can strengthen rhythms. Melatonin could well be one of these enhancers because it is an output of the circadian timing system that can also influence the activity of SCN neurons [41]. Such clock-driven entraining agents not only prevent rhythms from damping out in constant conditions, but may also tune the phase sensitivity of the circadian system. Melatonin increases the responsiveness of the circadian system through the activation of its receptors in the SCN [9]; it is also known that melatonin alters the firing rate of SCN neurons and speeds entrainment to a change in the Light/Dark cycle. In agreement with this idea, we observed that the shrews' rhythmic activity was reinforced each time a new implant was provided. Conversely, implanting aged animals that had lost rhythmic activity was ineffective (not shown). This would indicate that once disorganized, locomotor activity rhythm cannot be rescued; melatonin only serves to maintain an existing rhythm.

### Daily rhythm, Senescence, and natural selection

The short lifespan of *C. russula* (an average of 18 months in the wild [21]) is dramatically increased in captivity, where it frequently reaches 30 months. Laboratory conditions remove most of the extrinsic risks of mortality to which animals are exposed in the wild, and allows observation of their potential (intrinsic) lifespan [3]. With the exception of lifespan, all the major life-history traits keep the same timing for captive shrews compared to wild ones. Up to 12–14 mo, adult *C. russula* exhibit daily and seasonal rhythms in most of their biological functions [42], [43]. The first signs of aging appear at the same time in laboratory conditions as they do in the wild (arrhythmic daily locomotor activity and thermoregulation, absence of reproduction and erratic shedding [23]). Consequently, the adult phase remains the same in both situations, whereas the senescence period is increased in captivity. This result supports the disposable soma hypothesis [44]; the levels of the extrinsic rates of mortality drive the patterns of protection and repair mechanisms and may ultimately define lifespan. Hence, if shrews live in a wild environment where a specific set of constraints (resource availability, predation, parasitism) bring the expectation of living beyond 18 mo to zero, the development of a costly repair and protection mechanism that would keep the animal alive for much longer cannot be favored by natural selection. In captivity, even if animals are released from their natural environment, they will show protection from oxidative damage only for the “expected lifespan” (14–18 months) [3]. The free radical damage hypothesis postulates that a positive correlation exists between the rate of metabolism and oxidative damage, and supports the rate-of-living theory (the higher the energy expenditure, the shorter the lifespan) [20], [45]. Our continuous melatonin treatment obviously slowed the rate of aging of the diel locomotor activity rhythm. It is possible that the effect of melatonin reported in the present work results from the free radical scavenging properties of the hormone and its metabolites, which is referred as the free radical scavenging cascade [46]. Previous studies support evidence that aging is associated with an increase in free radical production and inflammation. In B6C3F1 mice, melatonin treatment restores a more youthful gene profile in aged animals' brains which, to a large extent involves reversal of age-induced elevation of basal inflammatory parameters [47]. Melatonin also counteracts the age-related production of cytokines and nitric oxide in senescence-accelerated mice [48]. Moreover, many tissues of old rats that were pinealectomized at an early age (which induces melatonin deficiency) show excessive oxidative damage compared to intact animals [49].

The physiological changes that aging shrews undergo in the wild are inimical to their survival. By decreasing body temperature and slowing down heart and respiratory movements, along with some other fundamental physiological functions, torpor allows adult healthy shrews to reduce their energy expenditure during daylight hours [50]. As circadian rhythms begin to dephase in aging animals, they lose the ability to enter torpor and thus increase daily energy expenditure. Moreover, being more active during the day exposes wild senescent shrews to a higher probability of predation, thus enhancing another component of the extrinsic mortality risk. And yet, we hypothesize that the negative effects caused by age-related behavioral disorders do not affect shrews' fitness: the loss of diel activity rhythms is concomitant with the loss of reproductive activity (Fons, 1988). Once the reproductive potential of an individual is over, one can assume that the maintenance of a coherent behavioral rhythm is no longer under selection.

### Conclusions

Shrews maintained in captivity exhibit a longer senescent phase by switching from the expected lifespan (which is determined by extrinsic factors in the wild) to an intrinsic or potential lifespan. Moreover, thanks to their unique physiology, shrews provide a portal through which we can discern the possible role of melatonin in aging: the hormone alone, under continuous administration, delays the appearance of one aspect of senescence, the loss of daily activity rhythm. The mechanisms of melatonin action in the implanted shrews and the physiological significance of our findings are among the crucial questions that remain to be answered. Melatonin could be acting either through receptor-based mechanisms [14] or by virtue of its antioxidant properties [11]. These results are of primary interest for understanding the effects melatonin has on the circadian system as well as on other processes affected by aging, and the use of melatonin as an anti-aging compound.

## Materials and Methods

### Animals

Wild White-toothed shrews *C. russula* were collected in the Banyuls-sur-Mer area (Pyrenées-Orientales, France) using non-baited pitfall traps. All the animals were transported to the Laboratoire Arago's animal facility. Shrews were housed in cages with 3 cm soil substratum, and provided with a hollow cylindrical piece of cork for shelter and moss for nesting material. All experimental procedures were approved by the Centre National de la Recherche Scientifique (CNRS).

### Melatonin Implants

The pharmacological dose of melatonin continuously delivered to individuals was supplied using implants adapted to the average body weight of shrews (10 g) [51]. They consisted of Silastic tubes (Dow Corning, medical grade, external diameter 1.96 mm, interior diameter 1.47 mm, length 5 mm) filled with 5 mg melatonin (N-acetyl 1-5-methoxytryptamine; Sigma Chemicals, St Louis, MO), and plugged with medical silicone (type A; Dow Corning, Midland, MI). One tube per animal was implanted subcutaneously in the dorsal region near the clavicle. Empty implants were prepared following the same protocol but as a separate set in order to avoid melatonin contamination.

### Measurements of plasma and pineal melatonin content

Plasma and pineal levels of melatonin were measured for the first generation of wild-trapped *shrews* ranging from 1 to 32 mo. Individuals were randomly assigned to a treatment (implanted with melatonin or not implanted), an age, and a light phase group. Animals were euthanized either during the night or during the day (4 pm or 11 pm UT respectively). A total of 57 control shrews born in captivity were sampled from 1 to 32 mo, both for plasma and pineal gland assays. Plasma levels of melatonin were also measured in 4 implanted animals, 1 week and 3 months (2 each) after implantation.

Pineal glands were flash frozen in liquid nitrogen immediately after collection, and stored at −80°C. In order to extract melatonin, they were defrosted and ground in 200 µL methanol using a sonicator (5 sec, Tmax = 40°C). The mix was completed with 300 µL methanol and homogenized by vortexing for 2 min. Samples were centrifuged for 2 min at 3,000 rpm at room temperature. The supernatant was then transferred to a new collection tube, and placed under high vacuum (1×10^−3^ Torr) until the liquid was completely removed. Samples were suspended in 50 µL ultra pure water, and stored at −20°C until the assay was performed. Blood was collected in heparin tubes and kept on ice for 4 hours. Samples were centrifuged for 10 min at 2,500 rpm at room temperature to extract the plasma and then stored at −80°C until use.

An indirect measurement was also performed in order to more accurately estimate the dose delivered by the implant over time. Six melatonin implants were individually immersed in 1 mL physiological buffer maintained at 37°C (shrews' body temperature), and renewed every day for 130 days. The melatonin content of the liquid was measured from day 1 to 7, 10 to 22, 30 to 48, and finally at days 59, 78, and 130.

Physiological buffer, plasma and pineal melatonin levels were measured using an enzyme-linked immunosorbent (ELISA) kit after purification according to the kit protocol (IBL, Hambourg). A nonparametric two-way analysis of variance (Scheirer-Ray-Hare test, [52]) was run using the R 2.8.1 package to assess the effect of light, age and the light*age interaction on pineal and plasma melatonin production. Mann Whitney U tests were then performed for pairwise comparison of means.

### Monitoring of shrews' activity rhythm

Juvenile (one to 2 mo) wild shrews were housed separately from mating pairs. By the age of nine to 10 months, they were transferred from a 12∶12 light∶dark (LD) cycle to a natural one. The ambient temperature was 22±2°C. They were housed in 50×30×70 cm individual glass *vivaria* where the floor was covered with soil; in the *vivaria* the animals were provided with an intricate light-proof plaster structure for shelter. Shrews were supplied *ad libitum* with water and fresh food consisting of crickets (*Acheta domesticus*) and mealworms (*Tenebrio molitor*). Water and food were supplied outside the nest and at a different time each day. The acclimation period to the daily activity monitoring system lasted at least 60 days before the beginning of the experiment. By the age of 12 months, male and female shrews were randomly assigned to three treatment groups consisting of animals that received a subcutaneous melatonin implant (seven shrews) whereas control shrews received either an empty implant or no implant at all (four animals total). The implants were renewed two times every five months. The sample size corresponded to 11, 9, and 6 shrews at, 12, 17 and 21 mo respectively. Only four individuals remained alive until 30 mo. Mortality was not significantly affected by the melatonin treatment (Mann-Whitney U test, adjusted Z = −1.15, *P* = 0.250). Recording the animal entrances and exits of the nest was performed using two infrared emitting diodes coupled to phototransistor sensors placed at the entrance of each plaster nest. The sensors were connected to a computer via a multiplexer. The data were acquired simultaneously and continuously (24 h a day, 7 days a week) for each individual of the three treatments for up to 535 days, and managed by generating a MySQL database.

### Analysis of rhythmic activity data

We used the khi-2 periodogram to define the activity rhythm periodicity. We looked for periods in the time series between 14 and 34 h. Ten day-long subseries were analyzed applying the moving window principle: successive subseries overlapping by one day were built and analyzed (sub-series 1: day 1 to day 10, subseries 2: from day 2 to day 11, etc [53]. This technique allowed us to follow the evolution of the periodicities present in the series. The significance of the 24 h period was displayed as a “24 h period degradation index”. This index was estimated by first calculating the *q(p)* statistic for *p* = 24 h:
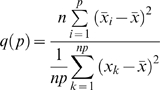
where *p* = period (24), *n* = row number (10), 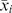 = mean number of exits for column i (10 values), *x_k_* = number of exits (for *k* = 1 to *np* = 240; [53] demonstrated that *q(p)* behaves as a khi-2 variable with p−1 degrees of freedom. A critical value 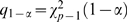 was therefore calculated where *1/p* corresponds to the “24 h-period significance index”.

An amplitude degradation index *I_(d)_* was also built in order to track the evolution of the amplitude. I(d) was obtained by calculating the mean activity profile on the first 30 days of the series and then by measuring the difference between this mean profile and each successive daily profile, according to the formula: 
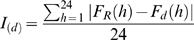
, where *d* = day, *h* = hour, *F_R_* = mean exit activity on the 30 first days for hour *h*, *F_d_* (h)  = exit activity at d day and h hour.

Activity profiles obtained for shrews that received an empty implant or no implant were pooled because the data obtained from the two groups did not differ significantly.
